# *Drosophila melanogaster* Imd signaling interacts with insulin signaling and alters feeding rate upon parasitic nematode infection

**DOI:** 10.1016/j.heliyon.2023.e16139

**Published:** 2023-05-18

**Authors:** Yaprak Ozakman, Dhaivat Raval, Ioannis Eleftherianos

**Affiliations:** a Infection and Innate Immunity Laboratory, Department of Biological Sciences, The George Washington University, Washington DC, 20052, USA

**Keywords:** *Drosophila*, Immunometabolism, *Heterorhabditis*, *Photorhabdus*, Insulin signaling

## Abstract

Significant progress has been made in recent years on exploring immunometabolism, a field that integrates two processes essential for maintaining tissue and organismal homeostasis, immunity and metabolism. The nematode parasite *Heterorhabditis gerrardi*, its mutualistic bacteria *Photorhabdus asymbiotica*, and the fruit fly *Drosophila melanogaster* constitute a unique system to investigate the molecular basis of host immunometabolic response to nematode-bacterial complexes. In this study, we explored the contribution of the two major immune signaling pathways, Toll and Imd, to sugar metabolism in *D. melanogaster* larvae during infection with *H. gerrardi* nematodes. We infected Toll or Imd signaling loss-of-function mutant larvae with *H. gerrardi* nematodes and assessed larval survival ability, feeding rate, and sugar metabolism. We found no significant differences in the survival ability or levels of sugar metabolites in any of the mutant larvae when responding to *H. gerrardi* infection. However, we found that the Imd mutant larvae have higher feeding rate than controls during the early stages of infection. In addition, feeding rates are lower in Imd mutants relative to the control larvae as the infection progresses. We further showed that *Dilp2* and *Dilp3* gene expression increases in Imd mutants compared to controls early in the infection, but their expression levels decrease at later times. These findings indicate that Imd signaling activity regulates the feeding rate and *Dilp2* and *Dilp3* expression in *D. melanogaster* larvae infected with *H. gerrardi*. Results from this study facilitate our understanding of the link between host innate immunity and sugar metabolism in the context of infectious diseases caused by parasitic nematodes.

## 1 Introduction

In recent years, considerable amount of research has been devoted to exploring immunometabolism, a field at the interface of two distinct yet principal areas in organismal well-being, immunity and metabolism [1]. The sum of biochemical reactions in living organisms that either result in production or consumption of energy defines metabolism, which impacts all cellular functions and plays a fundamental role in biological processes. Host immune responses against harmful microorganisms play essential roles in the regulation of metabolic processes in vertebrates. In addition, the metabolic state of an organism is a critical determinant of a functional immune system [[1], [2], [3]].

The fruit fly *Drosophila melanogaster* has long been accepted as a suitable model host to study infection and innate immunity. Many significant advances in the field of innate immunity have been made through studies in *D. melanogaster* due to the remarkable evolutionary conservation of the immune signaling pathways between flies and vertebrates [4,5]. In particular, the two major immune signaling pathways in flies*,* the Toll and immune deficiency (Imd) share significant similarities with the mammalian Toll like receptors (TLR) and tumor necrosis factor receptor (TNF-R) signaling, respectively [[6], [7], [8]]. Induction of the Imd signaling (mainly by Gram-negative bacteria), and Toll signaling (mainly by Gram-positive bacteria and fungi), leads to the transcriptional activation of antimicrobial peptides (AMPs), which act to prevent the proliferation and dispersal of invading pathogens [[9], [10], [11]]. Infection of *D. melanogaster* with entomopathogenic nematodes (EPNs) is also associated with the transcriptional activation of AMPs. In particular, *Heterorhabditis bacteriophora* infection of *D. melanogaster* larvae induces the transcriptional activation of *Attacin* and *Diptericin* (Imd pathway) and *Drosomycin* and *Metchnikowin* (Toll pathway) [12]. In addition, excreted secreted products isolated from *H. bacteriophora* nematodes induce *Diptericin* expression in *D. melanogaster* larvae and adult flies [13].

In addition to its use in immunity research, *D. melanogaster* has started to emerge as a valuable experimental paradigm to dissect the molecular basis of host metabolic response during infection [[14], [15], [16], [17], [18]]. Recent work has shown that infection of *D. melanogaster* adult flies with Zika virus disrupts lipid homeostasis by inducing the accumulation of enlarged lipid droplets [16]. In addition, *Listeria monocytogenes*, *Mycobacterium marinum* or *Photorhabdus luminescens* infection results in loss of metabolic stores, such as glycogen in adult flies [[19], [20], [21]]. Infection with the entomopathogenic nematodes (EPNs) *Steinernema carpocapsae*, *Heterorhabditis bacteriophora* or *H. gerrardi* is also associated with metabolic shifts in *D. melanogaster* larvae [14,15,22]. More precisely, while *S. carpocapsae* infection leads to the formation of larger lipid droplets, *H. gerrardi* infection leads to the formation of smaller lipid droplets in larvae [15,23].

Although several studies have illustrated the metabolic changes that take place in *D. melanogaster* during infection with viral, bacterial pathogens or nematode parasites, whether and how immune signaling pathway activity interacts with the metabolic response upon infection has yet to be elucidated [[14], [15], [16],^21^,^22^]. To this end, we characterized the interactions between Toll and Imd signaling activity and sugar metabolism particularly against infection with parasitic nematodes. For this, we used *D. melanogaster* larvae loss-of-function mutations in the transcription factors of the Toll (*Dif*) or Imd (*Relish*) signaling pathways and infected the mutant larvae with *H. gerrardi* nematodes containing mutualistic *Photorhabdus asymbiotica* bacteria [24]. Following infection with the nematode-bacteria pairs, we assessed the changes in larval survival ability, feeding rate, and sugar levels, and also determined the transcript levels of genes involved in insulin signaling.

Our results reveal that Toll and Imd signaling activities are not required for the survival response of *D. melanogaster* larvae to *H. gerrardi* infection. We also show that Imd signaling activity is associated with reduced feeding rate in larvae following *H. gerrardi* infection. We further find no interaction between *D. melanogaster* Toll and Imd signaling activity and the accumulation of trehalose, glucose or glycogen in the context of *H. gerrardi* nematode infection. However, our results indicate an association between the Imd and insulin signaling in *D. melanogaster* larval anti-nematode response through changes in the transcript levels of *Drosophila insulin-like peptides 2 (Dilp2)* and *(Dilp3).* These results provide important insight into the interactions between host immunity and metabolism for fighting infections with parasitic nematodes and thus may lead to the identification of new strategies for treating infectious diseases.

## 2 Materials and methods

### 2.1 Fly and nematode stocks

All *Drosophila melanogaster* flies were raised on *Drosophila* medium (Lab Express) with a few granules (approximately 0.003 g) baker's yeast (Carolina Biological Supply, Burlington, NC, USA) at 25 °C, and a 12:12-h light:dark photoperiodic cycle. Fly lines *Dif*^*1*^ and *Rel*^*e20*^ were obtained from Dr. Jean-Marc Reichhart's lab (National Center for Scientific Research, Strasbourg, France) [25]. Fly line *w*^*1118*^ (strain 3605, Bloomington, IL, USA) was used as background control for *Rel*^*e20*^ and fly line cn *bw* (gifted from Dr. Louisa Wu's lab, University of Maryland, College Park, USA) was used as background control for *Dif*^*1*^. Late second to early third instar larvae were used in the experiments except for the feeding assay where late second instar larvae only were used. Production of *Heterorhabditis gerrardi* nematodes in the larvae of the wax moth *Galleria mellonella* was described previously [14,26]. All nematodes were used within one to five weeks after collection.

### 2.2 Larval infection

Microtiter 96-well plates were used to infect *D. melanogaster* larvae with *H. gerrardi* infective juveniles (IJs). Each plate was prepared by adding 100 μL of 1.25% agarose in each well. Nematode suspension of 10 μL containing 100 IJs was added to each well along with a single larva. Sterile distilled water of 10 μL served as the uninfected control. Wells were then sealed with a transparent film (USA Scientific, Ocala, FL, USA) and two holes were pierced for aeration. Plates were stored in the dark at 25 °C for 12 or 36 h before processing the larvae for further experiments. Each infection experiment was repeated three times with new batches of larvae and nematodes.

### 2.3 Survival response

To assess the survival response of *D. melanogaster* larvae to *H. gerrardi* infection, 24 larvae of each line were infected with nematodes or treated with sterile water as negative control. Survival was monitored at 12 h intervals and up to 72 h after infection. Larvae that failed to respond to stimulation with a pipette tip were scored as dead. Three independent survival experiments were performed.

### 2.4 Gene transcript level analysis

To quantify the expression levels of candidate genes, total RNA was extracted from four to seven *D. melanogaster* larvae using the TRIzol™ reagent according to the manufacturer's instructions. Reverse transcription was carried out using an Applied Biosystems High-Capacity cDNA Reverse Transcription Kit according to manufacturer's instructions. Quantitative RT-PCR (qRT-PCR) experiments were performed in a CFX96 Real-Time System, C1000 Thermal Cycler (Bio-Rad) as previously described [14]. The list of gene specific primers used in this study can be found in Table 1. The amount of mRNA in each sample was normalized to mRNA values of the housekeeping gene *RpL32* and presented as a ratio of the value for infected larvae to that of the uninfected controls. Each experiment was run in technical triplicates and repeated three times.

**Table 1:** Primers and their sequences used in quantitative RT-PCR experiments.

Gene	Accession No	Primer (5′-3′)	Sequence	Tm (^o^C)
*RpL32*	CG7939	Forward Reverse	GATGACCATCCGCCCAGCA CGGACCGACAGCTGCTTGGC	60
*Dilp2*	CG8167	Forward Reverse	TCCACAGTGAAGTTGGCCC AGATAATCGCGTCGACCAGG	57
*Dilp3*	CG14167	Forward Reverse	AGAGAACTTTGGACCCCGTGAA TGAACCGAACTATCACTCAACAGTCT	59
*Dilp5*	CG33273	Forward Reverse	AGTTCTCCTGTTCCTGATCC CAGTGAGTTCATGTGGTGAG	57
*Dilp6*	CG14049	Forward Reverse	ATATGCGTAAGCGGAACGGT GCAAGAGCTCCCTGTAGGTG	57
*fOXO*	CG3143	Forward Reverse	AGGCGCAGCCGATAGACGAATTTA TGCTGTTGACCAGGTTCGTGTTGA	60

### 2.5 Feeding rate estimation

To measure the feeding rate, six late second instar *D. melanogaster* larvae from each line were infected with H. gerrardi IJs or treated with sterile distilled water and then collected at 12 or 36 h post-infection. Larvae were fed on dyed food containing 2.5% yeast extract (Sigma), 2.5% d-sucrose (Fisher Scientific), 1% FD&C Blue No1 Dye (Spectrum), and 1% Agar (Fisher Scientific) for 20 min, and rinsed in deionized water. Larvae were then homogenized in 250 μL of sterile deionized water and homogenates were centrifuged for 10 min following aspiration of the supernatants into a fresh set of tubes containing 50 μL of absolute ethanol. The tubes with the supernatants were vortexed for 30 s and centrifuged again for 10 min. Absorbance of the dye was detected as previously described [27]. Samples were loaded onto a 96-well micro plate and absorbance was measured at 633 nm using a plate reader (BioTek). The experiment was performed three times.

### 2.6 Measurement of trehalose, glucose, and glycogen levels

To determine the sugar content in *D. melanogaster* larvae, six individuals were collected at the 12- or 36-h time point following each treatment. To quantify glucose or glycogen levels, larvae were homogenized with a pellet pestle on ice in 100 μL of 1 × PBS. To quantify trehalose levels, larvae was homogenized with a pellet pestle on ice in 100 μL Trehalase buffer (TB; 5 mM Tris pH 6.6, 137 mM NaCl, 2.7 mM KCl) [22]. Following, homogenization, protein concentrations were measured using the Pierce™ BCA Protein Assay Kit (Thermo Fisher Scientific; 23227, Waltham, MA, USA).

### 2.7 Trehalose levels

Following the estimation of protein concentration, to measure the levels of trehalose, the samples were initially diluted 1:3 in TB, and then further diluted 1:1 in either TB or Trehalase Stock (TS; 3 μL of porcine trehalase in 1 mL of TB). Diluted samples were incubated at 37 °C for 24 h in a clear 96-well plate before 100 μL of hexokinase reagent (Glucose Assay Reagent, Sigma-Aldrich; G3293, St. Louis, MO, USA) were added to each well. Absorbance was measured at 340 nm using a plate reader (BioTek). To calculate trehalose levels, the amount of free glucose was subtracted from samples digested in TS.

### 2.8 Glucose and glycogen levels

Following protein quantification, to measure the levels of glucose and glycogen, the samples were first diluted 1:3 in PBS and then further diluted again 1:1 in either amyloglucosidase stock solution (1.5 μL of amyloglucosidase in 1 mL of PBS, Sigma-Aldrich) or PBS. Samples (30 μL) were subsequently incubated at 37 °C for 60 min in a clear 96-well plate and hexokinase reagent (100 μL) was added to each well. Following an additional incubation at room temperature for 15 min, absorbance was measured at 340 nm using a plate reader (BioTek). Glucose levels were calculated by a glucose standard curve. For glycogen, the absorbance of glucose was subtracted from the absorbance of samples diluted with amyloglucosidase stock.

The levels of trehalose, glucose, and glycogen were determined relative to the protein content in each sample. All experiments were repeated three times.

### 2.9 Statistical analysis

Data plotting and statistics were performed using the GraphPad Prism8 software. Statistical analysis of the data from the survival experiments was conducted using a log-rank (Mantel-Cox). Data from the rest of the experiments were statistically analyzed using one-way analysis of variance (ANOVA) with a Bonferroni correction.

## 3 Results

Toll and Imd signaling activities are not essential for the survival response of *D. melanogaster* larvae to *H. gerrardi* infection.

**Fig. 1:**
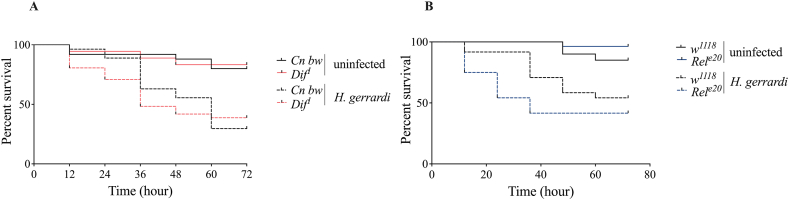
Survival of *Drosophila melanogaster Dif*^*1*^ (A) and *Rel*^*e20*^ (B) mutant larvae following *Heterorhabditis gerrardi* nematode infection. *Cn bw* is the background control line for *Dif*^*1*^mutants, while *w*^*1118*^ is the background control line for *Rel*^*e20*^ mutants. Treatment with water only served as the uninfected control. Larval survival was counted every 12 h and up to 72 h following infection. Significance differences in survival between the experimental treatments were assessed using Log-rank (Mantel-Cox) test and survival data are shown as Kaplan-Meier survival curves. (A) *Cn bw* and *Dif*^*1*^ infected with *H. gerarrdi* nematodes p = 0.8493 (B) *w*^*1118*^ and *Rel*^*e20*^ infected with *H*. *gerarrdi* nematodes p = 0.1534. Survival experiments were repeated three times and each experiment included biological duplicates for each fly line and treatment.

Inactivation of Toll and Imd signaling renders *D. melanogaster* adult flies more susceptible to infection with bacterial pathogens [28,29]. In addition, infection of *D. melanogaster Dif*^*1*^, *Rel*^*e20*^ single mutants and *Dif*^*1*^;*Rel*^*e20*^ double mutant adult flies with *H. gerrardi* or *H. bacteriophora* parasitic nematodes leads to higher mortality rates [30]. However, it is not known if Toll and Imd signaling activity contributes to the survival ability of *D. melanogaster* larvae upon parasitic nematode infection. To this end, we assessed the survival rate of *Dif*^*1*^ and *Rel*^*e20*^ mutant larvae following infection with the nematode parasite *H. gerrardi* (Fig. 1A–B). Contrary to the findings in adult flies, we did not observe any significant changes in the survival of any of the mutant larvae compared to their background controls upon nematode infection. These findings show that inactivating Toll or Imd signaling activity does not affect the survival phenotype of larvae when responding to *H. gerrardi* nematode infection.

Imd signaling activity reduces feeding rate in *D. melanogaster* larvae following *H. gerrardi* infection.

**Fig. 2:**
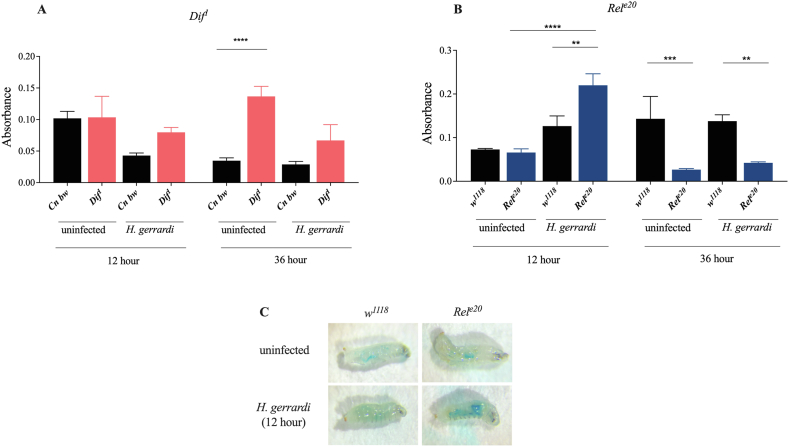
Quantification of the feeding rate in *Drosophila melanogaster* immune mutant larvae at 12 and 36 h post *Heterorhabditis gerrardi* nematode infection. *Cn b*w is the background control line for *Dif*^*1*^ mutant larvae, while *w*^*1118*^ is the background control line for *Rel*^*e20*^ mutants. (A) *Dif*^*1*^ 36 h ****p < 0.0001; (B) *Rel*^*e20*^ 12 h ****p < 0.0001, **p = 0.0025 and 36 h ***p = 0.0002, **p = 0.0020; (C) Feeding rate of *Rel*^*e20*^ mutant larvae at the 12-h time-point post *H. gerrardi* infection. Treatment with water only served as the uninfected control. Significance levels were assessed using one-way analysis of variance (ANOVA). The experiment was repeated three times with biological duplicates. Data represent means with standard deviation.

• The feeding rate can impact the survival ability of mammalian hosts at various levels and this effect can vary with the type of host and pathogen infection [31]. For instance in mice, high feeding rate accelerates the mortality rate against *Listeria monocytogenes* infection but promotes survival against influenza virus infection [32]. For this reason, we wanted to determine whether lack of mortality in *D. melanogaster* Toll and Imd signaling mutants after *H. gerrardi* infection is associated with changes in food consumption. To test this, we measured the feeding rate of *Dif*^*1*^, and *Rel*^*e20*^ mutant larvae and their background controls at 12 and 36 h post-infection (Fig. 2A–C). We selected 12 h as a relatively early time point based on the survival rates of the mutant larvae and 36 h as a later time point when nematode infection was established. We found that in the absence of infection, *Dif*^*1*^ mutants have increased feeding rate compared to their background controls. However, we did not observe any significant differences in the feeding rate between *Dif*^*1*^ mutants and *Cn bw* control larvae at either time point post nematode infection (Fig. 2A). Conversely, we found that *Rel*^*e20*^ mutant larvae have increased feeding rate compared to controls at 12 h post *H. gerrardi* infection (Fig. 2B). Visual representation of these results can also be seen in Fig. 2C. Interestingly, the feeding rates significantly decreased in *Rel*^*e20*^ mutants relative to their background controls at 36 h following infection. These results suggest that Imd but not Toll signaling activity regulates food consumption in *D. melanogaster* larvae during parasitic nematode infection, which is expressed as initial reduction during the early stages of infection followed by increase in feeding rate when the infection has progressed.

Toll and Imd signaling activity do not affect the levels of circulating or stored sugars in *D. melanogaster* larvae responding to *H. gerrardi* infection.

**Fig. 3:**
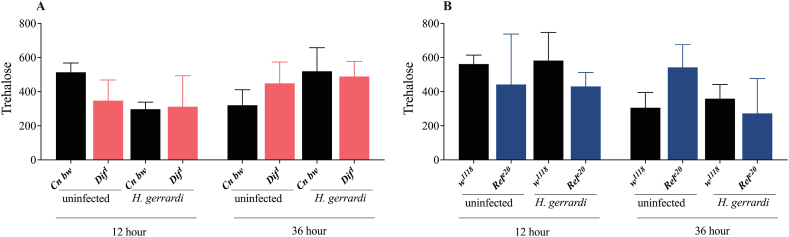
Trehalose levels (μg/ml) in *Drosophila melanogaster Dif*^*1*^ (A) and *Rel*^*e20*^ (B) mutant larvae at 12 and 36 h after *Heterorhabditis gerrardi* nematode infection. *Cn b*w is the background control line for *Dif*^*1*^ mutant larvae, while *w*^*1118*^ is the background control line for *Rel*^*e20*^ mutants. Treatment with water only served as the uninfected control. Significance levels were assessed using one-way analysis of variance (ANOVA). The experiment was repeated three times with biological duplicates. Data represent means with standard deviation.

**Fig. 4:**
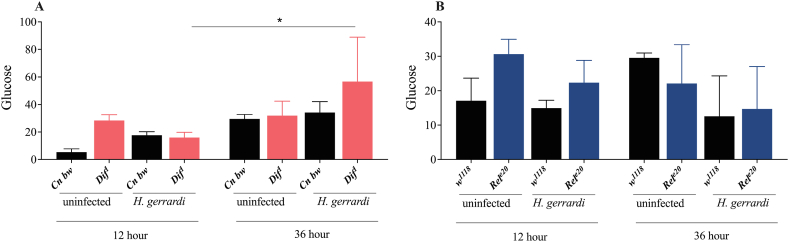
Glucose levels (μg/ml) in *Drosophila melanogaster Dif*^*1*^ (A) and *Rel*^*e20*^ (B) mutant larvae at 12 and 36 h post *Heterorhabditis gerrardi* nematode infection. *Cn b*w is the background control line for *Dif*^*1*^ mutant larvae, while *w*^*1118*^ is the background control line for *Rel*^*e20*^ mutants. (A) *p = 0.0342. Treatment with water only served as the uninfected control. Significance levels were assessed using one-way analysis of variance (ANOVA). The experiment was repeated three times with biological duplicates. Data represent means with standard deviation.

**Fig. 5:**
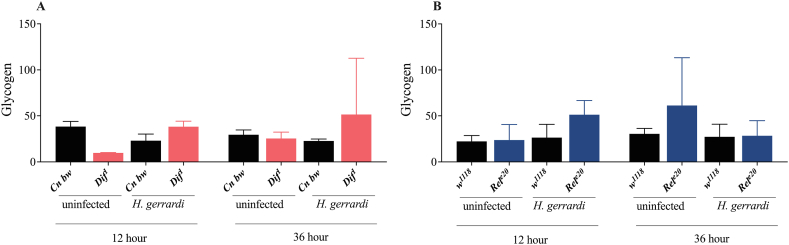
Glycogen levels (μg/ml) in *Drosophila melanogaster Dif*^*1*^ (A) and *Rel*^*e20*^ (B) mutant larvae at 12 and 36 h upon *Heterorhabditis gerrardi* nematode infection. *Cn b*w is the background control line for *Dif*^*1*^ mutant larvae, while *w*^*1118*^ is the background control line for *Rel*^*e20*^ mutants. Treatment with water only served as the uninfected control. Significance levels were assessed using one-way analysis of variance (ANOVA). The experiment was repeated three times with biological duplicates. Data represent means with standard deviation.

The Imd signaling pathway is a critical regulator of *D. melanogaster* sugar metabolism particularly in response to infection or inflammation [31]. For instance, in the absence of infection, reduced Imd signaling activity in glia leads to changes in the metabolic profile of adult flies, which include elevated levels of glucose and trehalose [33]. Ubiquitous inactivation of Imd signaling in *D. melanogaster* adults is also associated with impaired glucose tolerance in the absence of infection [18]. However, whether Toll or Imd signaling activity regulate the levels of circulating or stored sugars in *D. melanogaster* larvae during parasitic nematode infection remains unknown. To this end, we measured the levels of trehalose, glucose, and glycogen in *Dif*^*1*^ and *Rel*^*e20*^ mutant larvae at 12 and 36 h post-infection with *H. gerrardi* nematodes (Fig. 3A–B, Fig. 4A–B, Fig. 5A–B). We have not found any significant differences in trehalose levels between the *Dif*^*1*^ and *Rel*^*e20*^ mutant larvae and their corresponding background controls at either the 12 or 36 h time points post *H. gerrardi* infection (Fig. 3A–B). However, we observed a decrease in glucose levels in *Dif*^*1*^ mutant larvae (Fig. 4A) as the infection progressed. It is possible that persistent inflammation is causing the observed shifts in glucose stores over time. It is important to note that these results reflect the total levels of sugars in *D. melanogaster* and not the levels of glucose or trehalose in the hemolymph. Taken together, our results show that Toll and Imd signaling activities do not affect the levels of sugars in *D. melanogaster* larvae in the context of parasitic nematode challenge.

Imd signaling interacts with the insulin signaling via *Dilp2* and *Dilp3* expression in *H. gerrardi* infected *D. melanogaster* larvae.

**Fig. 6:**
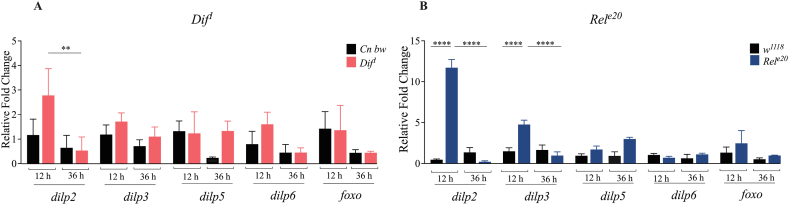
Transcriptional expression insulin signaling genes *Dilp2*, *Dilp3*, *Dilp5*, *Dilp6*, and *fOXO* in *Drosophila melanogaster Dif*^*1*^ (A) and *Rel*^*e20*^ (B) mutant larvae at 12 and 36 h upon *Heterorhabditis gerrardi* nematode infection. *Cn b*w is the background control line for *Dif*^*1*^ mutant larvae, while *w*^*1118*^ is the background control line for *Rel*^*e20*^ mutants. (A) **p = 0.0015; (B)****p < 0.0001. Treatment with water only served as the uninfected control. Significance levels were assessed using one-way analysis of variance (ANOVA). The experiment was repeated three times with biological duplicates. Data represent means with standard deviation.

In *D. melanogaster*, the insulin signaling is involved in a variety of processes from development and reproduction to metabolism and stress resistance [34,35]. *Previous* work has shown that insulin signaling is also involved in inflammation and antibacterial immunity in *D. melanogaster* through its interactions with the Imd signaling pathway [18,36]. For instance, constitutive activation of Imd signaling leads to reduced expression of the insulin signaling regulators, *Dilp3* and *Dilp6* in *D. melanogaster* larvae [18]*.* In addition, when the transcription factor of the insulin signaling, fOXO*,* is persistently activated in adult flies, expression of the peptidoglycan recognition protein SC2 (PGRP-SC2), negative regulator of the Imd signaling, is reduced [36]. To determine whether Toll and Imd signaling activity interacts with the insulin signaling in *D. melanogaster* larvae in the context of parasitic nematode infection, we quantified the transcript levels of genes regulated by the insulin signaling, including *Dilp2*, *Dilp3*, *Dilp5*, *Dilp6*, and *fOXO*, in *Dif*^*1*^ and *Rel*^*e20*^ mutant larvae at 12 and 36 h post-infection with *H. gerrardi* nematodes (Fig. 6). We have not observed significant differences in *Dilp* gene expression between *Dif*^*1*^ mutants and their background controls upon *H*. *gerrardi* infection (Fig. 6A). However, we found that *Dilp2* and *Dilp3* transcript levels were significantly higher in *Rel*^*e20*^ mutants compared to the *w*^*1118*^ background controls at 12 h post-infection. Interestingly, the transcript levels of *Dilp2* and *Dilp3* were lower in *Rel*^*e20*^ mutants at 36 h post-infection relative to the 12-h time point (Fig. 6B**)**. These results indicate that the Imd signaling interacts with the insulin signaling through decreasing the expression of *Dilp2* and *Dilp3* at the early stage of nematode infection. However, as the infection progresses, Imd signaling activity increases the expression of *Dilp2* and *Dilp3*. It is likely that Toll signaling does not interact with the insulin signaling in the context of parasitic nematode infection in *D. melanogaster* larvae.

## 4 Discussion

**Fig. 7:**
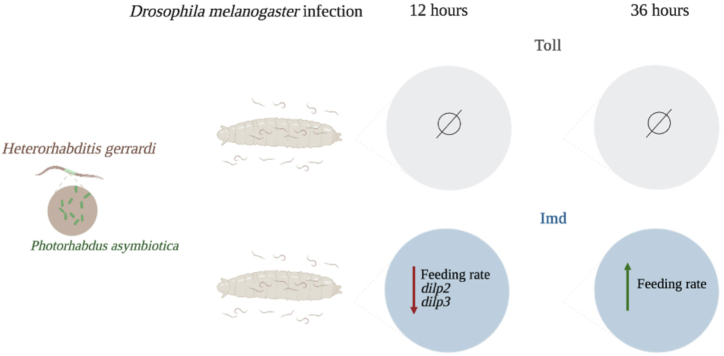
Proposed model of the interaction between immune signaling and feeding rate as well as insulin signaling in *Drosophila melanogaster* larvae responding to *Heterorhabditis gerrardi* infection. At 12-h post-nematode infection, Imd signaling activity reduces feeding rate and the expression of *dilp2* and *dilp3* genes, while at 36-h post-nematode infection, Imd signaling activity increases larval feeding rate. Toll signaling activity does not affect the feeding rate and the expression of *dilp* genes in nematode-infected larvae. The figure was created with BioRender.com.

In this study we have explored the contribution of the two Toll and Imd immune signaling pathways to regulating the survival ability, feeding rate, levels of sugar metabolites, and insulin signaling activity in *D. melanogaster* larvae upon infection with the parasitic nematode *H. gerrardi*. Our findings demonstrate that the *D. melanogaster* larval survival ability and levels of glucose, trehalose, and glycogen are not affected by Toll or Imd signaling activity upon *H. gerrardi* infection. However, we found that Imd signaling activity results in reduced feeding rate as well as changes in the transcript levels of insulin signaling components, *Dilp2* and *Dilp3* following *H. gerrardi* nematode infection (Fig. 7).

• Abnormal feeding rate is an indicator of sickness behavior and is conserved between vertebrates and invertebrates [32,37,38]. Infection induced changes in food consumption can regulate the *D. melanogaster* ability to combat infections. For instance, adult flies that become anorexic have increased tolerance against *Salmonella typhimurium* and decreased resistance against *L. monocytogenes* infection [38]. In addition, flies with reduced appetite show changes in AMP expression, such as decreased expression of the AMP genes *Drosomycin* and *Drosocin* and increased *Attacin* levels in response to *L. monocytogenes* infection [38]. Here, we show that following infection with the parasitic nematode *H. gerrardi*, Imd (*Rel*^*e20*^) mutant larvae have increased feeding rate compared to their background controls. Furthermore, feeding rates decrease significantly in *Rel*^*e20*^ mutants as the infection progresses. These results show that feeding rate is modulated by Imd signaling activity in *D. melanogaster* larvae responding to *H. gerrardi* infection. In contrast to Imd signaling mutants, there are no significant differences in feeding rate between *Dif*^*1*^ mutants and background control larvae following *H. gerrardi* infection. This indicates that Toll signaling activity is not involved in regulation of feeding rate to parasitic nematode infection. However, in the absence of infection *Dif*^*1*^ mutant larvae demonstrate an increased feeding rate compared to their background controls suggesting an interaction between *Dif* mediated Toll signaling and feeding capacity. In depth investigation of this potential interaction could be an interesting area for future studies.

In *D. melanogaster*, insulin signaling regulates a variety of processes including the metabolic response [34]. Dilps, hormones that bind to insulin receptors leading to the activation of the insulin signaling pathway, participate in the modulation of metabolic changes related to the activation of immune mechanisms [39]. In addition, Dilps orchestrate the action of metabolic organs such as fat body and gut, organs responsible for production of AMPs through the activation of Toll and Imd signaling [34,40,41]. Here we show that *Dilp2* and *Dilp3* transcript levels are significantly higher in loss-of-function mutants of Imd signaling mutants compared to their background controls during the early stage of *H. gerrardi* infection. However, as the infection progresses, the transcript levels of *Dilp2* and *Dilp3* decrease in Imd signaling mutants. Our findings suggest that Imd signaling activity regulates the expression of *Dilp2* and *Dilp3* to *H. gerrardi* infection. Similar to our findings at the early time point of infection with *H. gerrardi* nematodes, previous work has shown that constitutive activation of the Imd signaling leads to decreased levels of *Dilp3* [18]. It has been previously reported that persistent activation of the Toll signaling leads to suppression of insulin signaling activity [42]. Conversely, following infection with *H. gerrardi* nematodes there are no significant differences in the expression of *Dilps* or *fOXO* in loss-of-function mutants of Toll signaling compared to their background controls. Even though the interplay between Toll and insulin signaling in the absence of infection is evident, interaction between the two pathways is not observed in the context of parasitic nematode infection.

Taken together, results obtained from this study provide important insight into the contribution of the innate immune signaling pathways to regulating host sugar metabolism during infection with parasitic nematodes. In searching for novel and effective treatment strategies against parasitic nematode infection in humans*, D. melanogaster* is a great model system to reveal the molecular and functional factors that participate in the immunometabolic response to infection. In addition, with regard to their phylogenetic relationship, it is possible to make relevant comparisons between *Heterorhabditis* nematodes and vertebrate parasites which will potentially offer insight on the relationship between nematode parasitism and host immunometabolism [[43], [44], [45]].

## Author contribution statement

Yaprak Ozakman: designed and conducted the experiments, analyzed the data, constructed the figures, interpreted the results, and wrote drafts of the manuscript.

Dhaivat Raval: conducted parts of the experiments, analyzed the data, and contributed to a draft of the manuscript.

IOANNIS ELEFTHERIANOS: supervised the project, designed the experiments, interpreted the results, and revised the manuscript.

## Funding statement

This research did not receive any specific grant from funding agencies in the public, commercial, or not-for-profit sectors.

## Data availability statement

Data will be made available on request.

## Additional information

No additional information is available for this paper.

## Declaration of competing interest

The authors declare the following financial interests/personal relationships which may be considered as potential competing interests:

Ioannis Eleftherianos reports a relationship with The George Washington University that includes: employment.

## References

[1] Hotamisligil G.S. (2017). Foundations of immunometabolism and implications for metabolic health and disease. Immunity.

[2] Hotamisligil G.S. (2017). Inflammation, metaflammation and immunometabolic disorders. Nature.

[3] Lee Y.S., Wollam J., Olefsky J.M. (2018). An integrated view of immunometabolism. Cell.

[4] Hoffmann J.A. (2003). The immune response of *Drosophila*. Nature.

[5] Ugur B., Chen K., Bellen H.J. (2016). *Drosophila* tools and assays for the study of human diseases. Dis. Model Mech..

[6] Goberdhan D.C.I., Wilson C. (2003). The functions of insulin signaling: size isn't everything, even in *Drosophila*. Differentiation.

[7] Valanne S., Wang J.-H., Rämet M. (2011). The *Drosophila* Toll signaling pathway. J. Immunol..

[8] Myllymäki H., Valanne S., Rämet M. (2014). The *Drosophila* imd signaling pathway. J. Immunol..

[9] Michel T., Relchhart J.M., Hoffmann J.A., Royet J. (2001). *Drosophila* Toll is activated by Gram-positive bacteria through a circulating peptidoglycan recognition protein. Nature.

[10] Kleino A., Silverman N. (2014). The *Drosophila* IMD pathway in the activation of the humoral immune response. Dev. Comp. Immunol..

[11] Imler J.L., Bulet P. (2005). Antimicrobial peptides in *Drosophila*: structures, activities and gene regulation. Chem. Immunol. Allergy.

[12] Hallem E.A., Rengarajan M., Ciche T.A.A., Sternberg P.W. (2007). Nematodes, bacteria, and flies: a tripartite model for nematode parasitism. Curr. Biol..

[13] Kenney E., Hawdon J.M., O'Halloran D., Eleftherianos I. (2019). *Heterorhabditis bacteriophora* excreted-secreted products enable infection by *Photorhabdus luminescens* through suppression of the imd pathway. Front. Immunol..

[14] Ozakman Y., Eleftherianos I. (2019). TGF-β signaling interferes with the *Drosophila* innate immune and metabolic response to parasitic nematode infection. Front. Physiol..

[15] Yadav S., Frazer J., Banga A., Pruitt K., Harsh S., Jaenike J. (2018). Endosymbiont-based immunity in *Drosophila melanogaster* against parasitic nematode infection. PLoS One.

[16] Harsh S., Ozakman Y., Kitchen S.M., Paquin-Proulx D., Nixon D.F., Eleftherianos I. (2018). Dicer-2 regulates resistance and maintains homeostasis against Zika virus infection in *Drosophila*. J. Immunol..

[17] Ozakman Y., Eleftherianos I. (2020). Immune interactions between *Drosophila* and the pathogen *Xenorhabdus*. Microbiol. Res..

[18] Davoodi S., Galenza A., Panteluk A., Deshpande R., Ferguson M., Grewal S. (2019). The immune deficiency pathway regulates metabolic homeostasis in *Drosophila*. J. Immunol..

[19] Chambers M.C., Song K.H., Schneider D.S. (2012). *Listeria monocytogenes* infection causes metabolic shifts in *Drosophila melanogaster*. Freitag NE. PLoS One.

[20] Dionne M.S., Pham L.N., Shirasu-Hiza M., Schneider D.S. (2006). Akt and foxo dysregulation contribute to infection-induced wasting in *Drosophila*. Curr. Biol..

[21] Shokal U., Kopydlowski H., Harsh S., Eleftherianos I. (2018). Thioester-containing proteins 2 and 4 affect the metabolic activity and inflammation response in *Drosophila*. Infect. Immun..

[22] Ozakman Y., Pagadala T., Raval D., Eleftherianos I. (2020). The *Drosophila melanogaster* metabolic response against parasitic nematode infection is mediated by TGF-β signaling. Microorganisms.

[23] Ozakman Y., Eleftherianos I. (2019). TGF-Β signaling interferes with the *Drosophila* innate immune and metabolic response to parasitic nematode infection. Front. Physiol..

[24] Plichta K.L., Joyce S.A., Clarke D., Waterfield N., Stock S.P. (2009). *Heterorhabditis gerrardi* n. sp. (Nematoda: Heterorhabditidae): the hidden host of *Photorhabdus asymbiotica* (Enterobacteriaceae: γ-Proteobacteria). J. Helminthol..

[25] Hedengren M., Åsling B., Dushay M.S., Ando I., Ekengren S., Wihlborg M. (1999). Relish, a central factor in the control of humoral but not cellular immunity in *Drosophila*. Mol. Cell..

[26] White G.F. (1927). A method for obtaining infective nematode larvae from cultures. Science.

[27] Edgecomb R.S., Harth C.E., Schneiderman A.M. (1994). Regulation of feeding behavior in adult *Drosophila melanogaster* varies with feeding regime and nutritional state. J. Exp. Biol..

[28] Rutschmann S., Kilinc A. (2021).

[29] Lemaitre B., Kromer-Metzger E., Michaut L., Nicolas E., Meister M., Georgel P. (1995). A recessive mutation, immune deficiency (imd), defines two distinct control pathways in the *Drosophila* host defense. Proc. Natl. Acad. Sci. USA.

[30] Patrnogic J., Heryanto C., Ozakman Y., Eleftherianos I. (2019). Transcript analysis reveals the involvement of NF-κB transcription factors for the activation of TGF-β signaling in nematode-infected *Drosophila*. Immunogenetics.

[31] Galenza A., Foley E. (2019). Immunometabolism: insights from the *Drosophila* model. Dev. Comp. Immunol..

[32] Wang A, Huen SC, Luan HH, Gallezot J, Booth CJ, Medzhitov R, et al. Opposing effects of fasting metabolism on tissue tolerance in bacterial and viral inflammation article opposing effects of fasting metabolism on tissue tolerance in bacterial and viral inflammation Cell. 166: 1512-1518.e12. doi:10.1016/j.cell.2016.07.026. 10.1016/j.cell.2016.07.026 PMC5555589 27610573

[33] Kounatidis I., Chtarbanova S., Cao Y., Hayne M., Jayanth D., Ganetzky B. (2017). NF-κB immunity in the brain determines fly lifespan in healthy aging and age-related neurodegeneration. Cell Rep..

[34] Semaniuk U., Piskovatska V., Strilbytska O., Strutynska T., Burdyliuk N., Vaiserman A. (2021). *Drosophila* insulin‐like peptides: from expression to functions – a review. Entomol. Exp. Appl..

[35] Teleman A.A. (2010). Molecular mechanisms of metabolic regulation by insulin in *Drosophila*. Biochem. J..

[36] Guo L., Karpac J., Tran S.L., Jasper H. (2014). PGRP-SC2 promotes gut immune homeostasis to limit commensal dysbiosis and extend lifespan. Cell.

[37] Adamo S.A. (2005). Parasitic suppression of feeding in the tobacco hornworm, *Manduca sexta*: parallels with feeding depression after an immune challenge. Arch. Insect Biochem. Physiol..

[38] Ayres J.S., Schneider D.S. (2009). The role of anorexia in resistance and tolerance to infections in Drosophila. PLoS Biol..

[39] Dolezal T., Krejcova G., Bajgar A., Nedbalova P., Strasser P. (2019). Molecular regulations of metabolism during immune response in insects. Insect Biochem. Mol. Biol..

[40] Broderick N.A. (2016). Friend, foe or food? Recognition and the role of antimicrobial peptides in gut immunity and *Drosophila*-microbe interactions. Philos. Trans. R. Soc. B Biol. Sci..

[41] Buchon N., Silverman N., Cherry S. (2014). Immunity in *Drosophila melanogaster*–from microbial recognition to whole-organism physiology. Nat. Rev. Immunol..

[42] DiAngelo J.R., Bland M.L., Bambina S., Cherry S., Birnbaum M.J. (2009). The immune response attenuates growth and nutrient storage in *Drosophila* by reducing insulin signaling. Proc. Natl. Acad. Sci. U. S. A..

[43] Sommer R.J., Streit A. (2011). Comparative genetics and genomics of nematodes: genome structure, development, and lifestyle. Annu. Rev. Genet..

[44] Bai X., Adams B.J., Ciche T.A., Clifton S., Gaugler R., Kim K. (2013). A lover and a fighter: the genome sequence of an entomopathogenic nematode *Heterorhabditis bacteriophora*. PLoS One.

[45] Plotkin S., Diemert D.J., Bethony J.M., Hotez P.J. (2008). Hookworm Vaccines. Clin Infect Dis..

